# HMGB1 in the pathogenesis of ultraviolet-induced ocular surface inflammation

**DOI:** 10.1038/cddis.2015.199

**Published:** 2015-08-27

**Authors:** S J Han, H J Min, S C Yoon, E A Ko, S J Park, J-H Yoon, J-S Shin, K Y Seo

**Affiliations:** 1 Department of Ophthalmology, Institute of Vision Research, Severance Hospital, Yonsei University College of Medicine, Seoul, Korea; 2 Department of Otorhinolaryngology - Head and Neck Surgery, Chung-Ang University College of Medicine, Seoul, Korea; 3 Brain Korea 21 Plus Project for Medical Science, Yonsei University College of Medicine, Seoul, Korea; 4 Department of Microbiology, Yonsei University College of Medicine, Seoul, Korea; 5 Department of Otorhinolaryngology, Severance Hospital, Yonsei University College of Medicine, Seoul, Korea; 6 Severance Biomedical Science Institute and Institute for Immunology and Immunological Diseases, Yonsei University College of Medicine, Seoul, Korea

## Abstract

High-mobility group box 1 (HMGB1) functions as a transcription-enhancing nuclear protein as well as a crucial cytokine that regulates inflammation. This study demonstrated that secretion of HMGB1 due to ultraviolet (UV) radiation inducing ocular surface inflammation-mediated reactive oxygen species (ROS) production. After treating conjunctival epithelial cells with UV radiation, HMGB1 was translocated from the nucleus to the cytoplasm and then eventually to the extracellular space. HMGB1 played a crucial role in UV-induced conjunctival neutrophil infiltration, which subsided when mice were pretreated with the HMGB1 inhibitors soluble receptor for advanced glycation endproducts (sRAGEs) and HMGB1 A box protein. In case of using ROS quencher, there was decrease in UV-induced HMGB1 secretion in conjunctival epithelial cells and mice. Considering that UV-induced chronic inflammation causes ocular surface change as pterygium, we have confirmed high HMGB1 translocation and ROS expression in human pterygium. Our findings therefore revealed a previously unknown mechanism of UV-induced ocular inflammation related to ROS and HMGB1 suggesting a new medical therapeutic target.

High-mobility group box 1 (HMGB1) is a nuclear protein that binds to DNA functioning as a DNA chaperone.^1^ Various conditions such as acetylation, phosphorylation, or oxidation cause posttranslational modification of HMGB1 while influencing the location and action of HMGB1.^2, 3, 4, 5^ Because HMGB1 lacks a leader sequence, it can be transferred to the extracellular space by a vesicular transport mechanism known as the non-classical secretory pathway.^6, 7^ HMGB1 can also be passively released after necrosis or late apoptosis as a consequence of decreased plasma membrane permeability.^3, 8, 9^ In the extracellular environment, HMGB1 interacts with various receptors including Toll-like receptor (TLR)-2, -4, receptor for advanced glycation endproducts (RAGEs), or CD24-Siglec-10 in order to deliver signals like an alarmin.^10, 11^ In addition, HMGB1 is reported to act as a late mediator of endotoxemia and sepsis in animal models as well as human patients through interactions with TLR4.^12, 13^

The recruitment of inflammatory cells can be mediated through interactions among HMGB1, RAGE, and CXCR4,^14^ wherein HMGB1 functions as a signaling molecule during cell differentiation and migration in the extracellular space.^15, 16^ Endogenous HMGB1 translocates from the nucleus to the cytoplasm through a redox-dependent mechanism. In addition, cytoplasmic HMGB1 plays a role as a regulator between macroautophagy and apoptosis.^17^ HMGB1 contains two folded DNA-binding subunits known as A and B boxes along with an acidic region. The HMGB1 A box has anti-inflammatory effect whereas the B box has pro-inflammatory effect by inducement in releasing various cytokines. The purified recombinant A box functions as an antagonist to B-box-induced cytokine production.^18^

HMGB1 is also reported to have an association with numerous immune-mediated conditions, such as rheumatoid arthritis, systemic lupus erythematosus (SLE), or inflammatory myositis.^19, 20, 21^ Under such conditions, the expression of HMGB1 increases in the blood, synovial tissue, skin lesions, and especially in the cytoplasm as well as extracellular area of patient specimens.^19, 20, 21^ Ultraviolet (UV) radiation triggers cutaneous lupus erythematosus (CLE), and large amount of extracellular HMGB1 was observed in skin lesions of lupus erythematosus patients^20^ suggesting that UV radiation might cause the translocation of HMGB1.

Conjunctiva surrounding an eye is also exposed to outer environment like the skin does, and UV functions as its stimuli. Increased UV radiation on ocular surface elevates the level of oxidative stress causing induction of proteins, such as survivin or p53, on ocular surface.^22^ Direct effects of both UV radiation and UV-induced oxidative-free radicals can cause molecular alterations in the p53, p63, and p73 genes, which, in turn, can lead to alterations in cellular differentiation and cell cycle on conjunctiva.^23, 24, 25^ Alteration on ocular surface caused by UV results chronic inflammation, which is the main pathogenesis of pterygium.^26, 27^ Indeed, high prevalence of pterygium was noted in the equatorial zone of the tropics located within 30 degrees toward both North and South.^28^

On the basis of the previous findings regarding the association between cytoplasmic and secreted HMGB1 protein and UV-related inflammatory diseases, we invested the involvement of HMGB1 in chronic ocular surface inflammation. In this study, we found that the level of HMGB1 and reactive oxygen species (ROS) are increased in human pterygial tissues. Moreover, we confirmed that UV exposure and UV-induced oxidative stress could cause nucleo-cytoplasmic translocation of HMGB1 followed by its extracellular secretion using conjunctival epithelial cells. In a mouse model, HMGB1 played a critical role in chronic inflammatory cell recruitment to the UV-irradiated area. Pretreatment of HMGB1 neutralizing proteins or ROS scavengers could block the chemotaxis of inflammatory cells suggesting that HMGB1 might be a possible therapeutic target for the treatment of UV-induced pterygium formation.

## Results

### UV induces the nucleo-cytoplasmic translocation of HMGB1 that results in secretion from conjunctival cells

**Figure 1:**
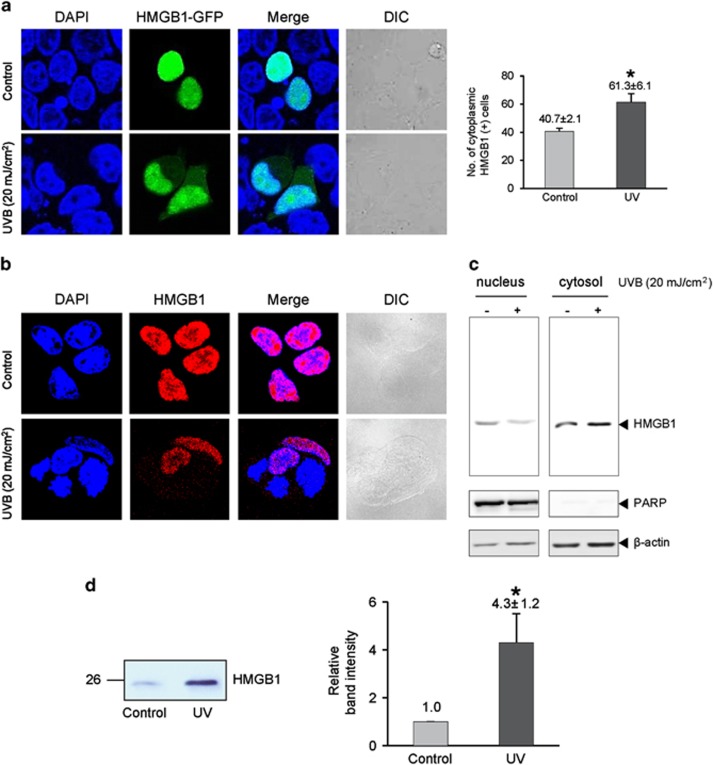
UV-induced nucleo-cytoplasmic translocation of HMGB1 in human conjunctival epithelial Chang cells. (**a**) Chang cells were transiently transfected with an HMGB1-GFP plasmid and then irradiated with 20 mJ/cm^2^ of UV followed by 8-h incubation. The number of cytoplasmic GFP-positive cells was counted among the 100 GFP-positive cells. (**b** and **c**) An immunofluorescence assay was performed to observe the UV-induced translocation of endogenous HMGB1. Endogenous HMGB1 was immunostained using an anti-HMGB1 antibody and a secondary anti-rabbit Alexa Fluor 594 antibody. Cytosolic proteins were fractionated from the lysates, and a western blot assay was performed. (**d**) Chang cells were irradiated with 20 mJ/cm^2^ of UV and incubated for further 8 h. The HMGB1 level in the culture supernatant was then evaluated by western blot. Relative band intensity was measured using the ImageJ program. Values shown are mean±S.E. *P<0.05 (*n*=3)

To observe whether UV-induced HMGB1 secretion is associated with the inflammation that occurs during pterygium development, we first observed whether UV radiation could induce the nucleo-cytoplasmic translocation of HMGB1 in human conjunctival Chang cells. Chang conjunctival cells were transiently transfected with an HMGB1-GFP plasmid followed by irradiation with 20 mJ/cm^2^ of UV, which induced no significant necrotic cell death in conjunctival cells (data not shown). However, we found translocation of HMGB1 to the cytoplasm using confocal microscopy (Figure 1a). To observe the endogenous HMGB1 movement, an immunofluorescence assay was performed. As shown in Figure 1b, the translocation of endogenous HMGB1 was observed after UV treatment by confocal microscopy and western blot analysis of the cytosolic fraction of UV-treated Chang cells (Figure 1c). We then examined if UV-induced secretion of HMGB1 to the extracellular area had occurred. The results showed that the level of HMGB1 in the culture supernatant of Chang cells was significantly increased 8 h after the UV radiation (Figure 1d). We assessed mRNA expression levels of cytokines in human conjunctiva epithelial Chang cells in a time-dependent manner. We found that MIP-1, IL-17, IL-8, and HMGB1 were increased after the irradiation. At 7 h after exposure to UVB light, the expression levels of MIP-1, IL-17, and IL-8 reached maximum, while the HMGB1 levels were evaluated at 8 h after UVB irradiation (Supplementary Figure S1).

### UV-induced translocation of HMGB1 is inhibited by antioxidants

**Figure 2:**
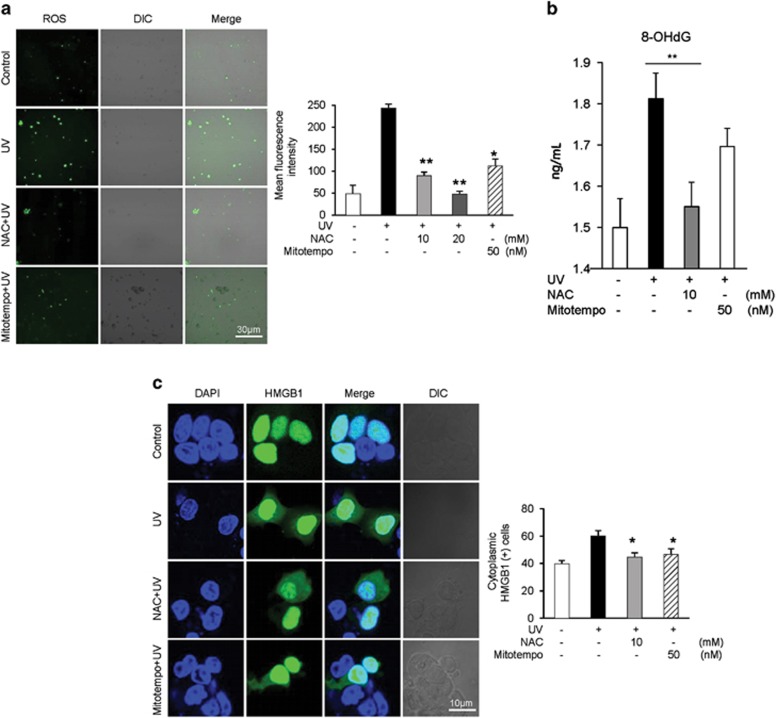
UV-induced ROS generation resulting in the translocation of HMGB1. (**a**) Chang cells were treated with 20 mJ/cm^2^ of UV with or without pretreatment with NAC and Mito-Tempo (Enzo Life Sciences). Approximately, 5 *μ*mol/l of DC-FDA was used to detect ROS generation. Mean fluorescence intensity was measured using FV1000 confocal microscopy. *P<0.05 and **P<0.01 (*n*=3). (**b**) Chang cells were treated with 20 mJ/cm^2^ of UV with or without pretreatment with NAC and Mito-Tempo (Enzo Life Sciences). At 8 h after UV treatment, 8-OHdG levels were measured by ELISA. **P<0.01 (*n*=3). (**c**) Chang cells were transfected with the HMGB1-GFP plasmid and incubated for 24 h. UV treatment was performed with or without pretreatment with NAC (10 mM) and Mito-Tempo (Enzo Life Sciences, 50 nM) 1 h before UV radiation. After 8 h, the GFP-tagged HMGB1 signal was visualized by confocal microscopy. The number of cytoplasmic HMGB1-GFP cells was then counted. Values shown are the mean±S.E. *P<0.05 (*n*=3)

UV induces ROS generation while activating oxidative mechanisms.^29, 30^ We therefore asked whether UV-induced ROS generation occurred in Chang cells and if treatment with antioxidants such as *N*-acetyl-l-cysteine (NAC) and Mito-Tempo (Enzo Life Sciences, Farmingdale, NY, USA) could reduce UV-induced ROS generation and HMGB1 translocation. NAC is a precursor of glutathione that acts as a scavenger for OH radicals, and Mito-Tempo (Enzo Life Sciences) is a mitochondria-targeted antioxidant with superoxide and alkyl radical scavenging properties. The results presented that UV induced significant ROS formation, which was reduced by NAC and Mito-Tempo (Enzo Life Sciences) treatment (Figure 2a). The 8-OHdG level, one of the markers of superoxide production, was significantly increased in Chang cells after UV irradiation. NAC treatment did significantly reduce the 8-OHdG level, but Mito-Tempo (Enzo Life Sciences) treatment was slightly reduced (*P*=0.069; Figure 2b). Not surprisingly, we observed HMGB1 translocation in Chang cells inhibited by the antioxidants (Figure 2c); oxidative potential is an important factor in determining the location and function of HMGB1.^5, 31, 32^

### sRAGE and HMGB1 A box protein inhibit UV-induced leukocyte infiltration in mouse conjunctiva

**Figure 3:**
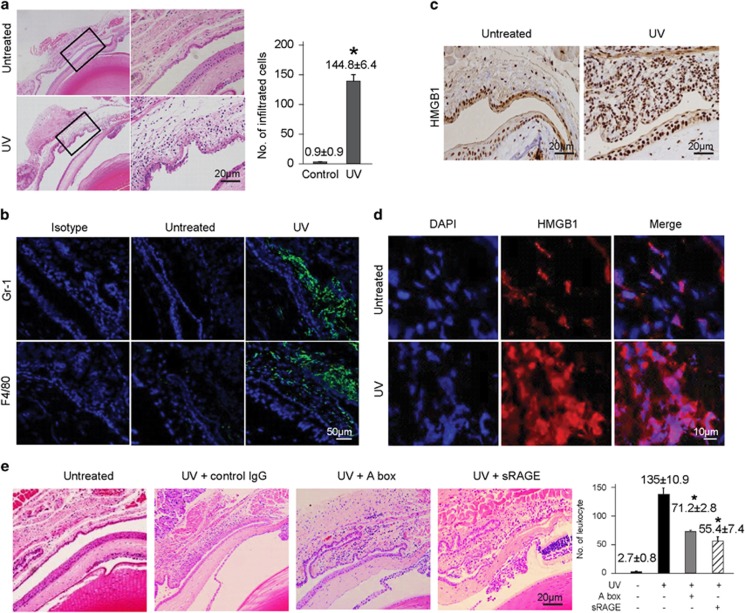
UV-induced leukocyte infiltration was inhibited in mouse conjunctiva by HMGB1 A box and sRAGE treatments. (**a** and **b**) The conjunctiva of BALB/c mice were exposed to UV (311 nm) at a single dose of 100 mJ/cm^2^ using a UV lamp 24 h after UV treatment. The whole eye was then enucleated for H&E staining, and the number of infiltrated cells was counted. *P<0.05 (*n*=10). (**b**) A representative conjunctival tissue was immunofluorescently stained against Gr-1 (green, upper) or F4/80 (green, lower) for infiltrated leukocytes. DAPI was used to stain the nuclei. (**c**) Immunohistochemical staining of UV-exposed conjunctival tissue using an anti-HMGB1 antibody. (**d**) An immunofluorescence assay was performed to observe UV-induced nucleo-cytoplasmic translocation of endogenous HMGB1. (**e**) BALB/c mice were pretreated with sRAGEs (100 *μ*g per mouse, 15 min before UV radiation) and HMGB1 A box protein (150 *μ*g per injection, 1 h before and after UV radiation), and then treated with a single dose of 100 mJ/cm^2^ UVB. At 24 h after UV treatment, the whole eyes were enucleated for H&E staining and the infiltrated leukocyte cells were counted. Values shown are the mean±S.E. *P<0.05 (*n*=10)

We evaluated the effect of UV on HMGB1-induced leukocyte infiltration in BALB/c mice. The conjunctiva of mice were exposed to UV (single dose of 100 mJ/cm^2^) followed by the whole eye removal 24 h after UV irradiation for immunohistochemical analysis. UV radiation induced the infiltration of cells to the mouse conjunctiva (Figure 3a), which predominantly were composed with Gr-1^+^ or F4/80^+^ cells (Figure 3b). The HMGB1 expression level was also elevated in conjunctival epithelial cells (Figure 3c), and the HMGB1 protein was translocated to the cytoplasm (Figure 3d), implying the secretion of HMGB1. To verify whether UV-induced inflammation was associated with HMGB1, we pretreated BALB/c mice with sRAGE and HMGB1 A box proteins, which are a well-known soluble decoy receptor of HMGB1 and an inhibitory domain of HMGB1, respectively,^18^ followed by irradiation with UV. Pretreatment with sRAGEs and the A box of HMGB1 significantly reduced UV-induced cell infiltration (Figure 3e). These results indicate that UV radiation after conjunctival inflammation is highly associated with HMGB1 secretion.

### *In vivo* treatment of antioxidants reduces UV-induced infiltration of inflammatory cells in mouse conjunctiva

**Figure 4:**
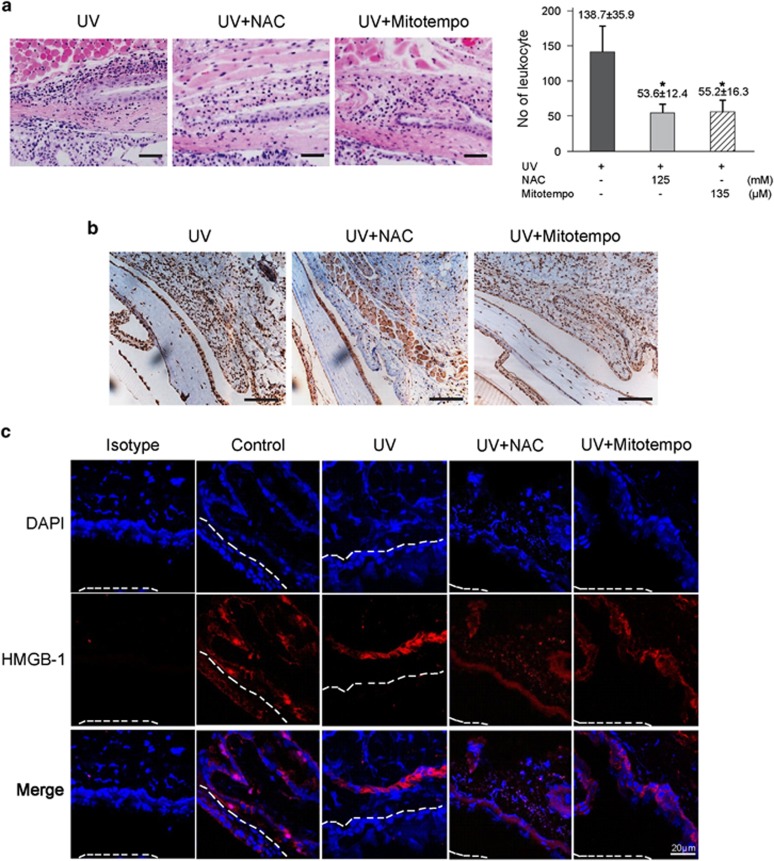
Antioxidant treatment reduced UV-induced infiltration of inflammatory cells to the conjunctiva. BALB/c mice were intraperitoneally treated with NAC (125 mM, *n*=5) and Mito-Tempo ( Enzo Life Sciences, 135 *μ*M, *n*=5) and then exposed to 10 mJ/cm^2^ of UVB. The eyes were enucleated on 24 h after UV treatment. (**a**) Histologic analysis was performed with H&E staining. The number of leukocyte cells was counted and the mean±S.E. was obtained. *P<0.05. (**b**) Immunohistochemical staining of the eye tissue using an anti-HMGB1 antibody. (**c**) Immunofluorescence confocal microscopy was performed with monoclonal antibody of HMGB1 (red) and DAPI (blue). Surface of the cornea epithelium (dotted line)

We investigated whether antioxidant treatment could inhibit the infiltration of leukocytes in UV-treated mice. BALB/c mice were treated with a single dose of 100 mJ/cm^2^ UVB after pretreatment with NAC (125 mM, 2 h before UV) or Mito-Tempo (Enzo Life Sciences, 135 *μ*M, 24 h and 1 h before UVB). At 24 h after UV radiation, whole eyes were enucleated and observed for the infiltration of leukocytes by histological analysis. NAC and Mito-Tempo (Enzo Life Sciences) pretreatment significantly reduced UV-induced infiltration of leukocytes to the mouse conjunctiva (Figures 4a and b). We conducted confocal microscopy in order to clearly confirm that HMGB1 release in UV-induced inflammation can be controlled with NAC and Mito-Tempo (Enzo Life Sciences). We could find out clearer HMGB1 translocation after the UV irradiation, and there was decrease in HMGB1 staining when ROS quencher was pretreated (Figure 4c). These results suggest that UV-induced ROS facilitates HMGB1 secretion, which is involved in the regulation of inflammatory responses on the ocular surface.

### Nucleo-cytoplasmic translocation of HMGB1 in human pterygial tissue

**Figure 5:**
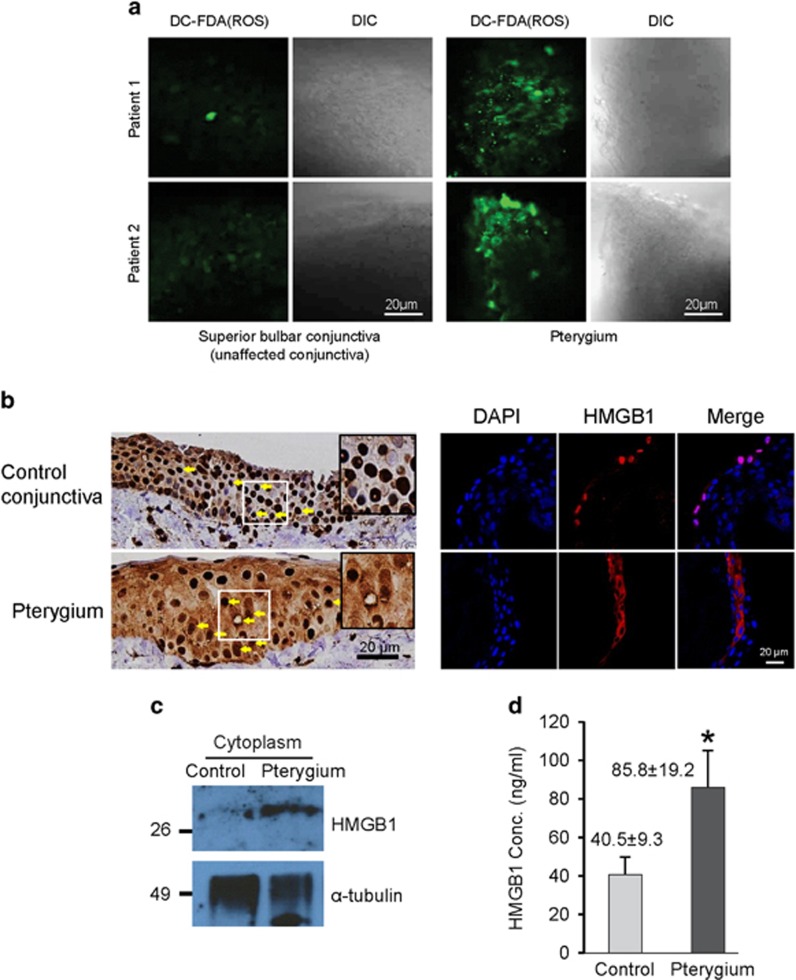
ROS production and cytoplasmic HMGB1 increased in conjunctival tissue from pterygium patients. (**a**) Two pterygium conjunctival tissues were excised and incubated with 5 *μ*M of DC-FDA in culture medium. ROS levels were measured by FV1000 confocal microscopy. ROS levels were elevated in the two pterygium conjunctival tissues compared to the superior bulbar conjunctival tissues from the same two patients. (**b**) Human pterygial tissues were surgically resected from five patients, and immunohistochemical (left) and immunofluorescence (right) assays were performed for detecting HMGB1. Control conjunctival tissues were obtained from the superior bulbar conjunctiva of the same patients. (**c**) Cytosolic proteins were fractionated from the pterygium (P) and control (C) conjunctiva lysates followed by a western blot assay. (**d**) Secretory HMGB1 levels in the culture supernatant were determined by ELISA and means±S.E. are shown. *P<0.05 (*n*=5)

A pterygium is a benign, invasive, proliferative disease on conjunctiva, which is induced as chronic inflammation^26, 27^ resulting connective tissue remodeling. We first tested whether the pterygium conjunctival tissue produced ROS because pterygium development is correlated to the amount of exposure to the sunlight.^28^ ROS was measured from the conjunctival tissues obtained from two pterygium patients and their corresponding superior bulbar conjunctival tissues as controls. Superior bulbar conjunctival tissue is rarely exposed to UV ray since it is usually covered with the eyelid. Pterygium conjunctival tissues had high level of ROS expression compared to the control conjunctiva (Figure 5a). Since the increase in ROS causes oxidative stress followed by the translocation of HMGB1,^33^ we investigated whether human conjunctival tissues from pterygium exhibit cytoplasmic translocation of HMGB1. Human conjunctival tissue was surgically obtained from the pterygium and superior bulbar conjunctiva of five patients followed by immunochemical stain for HMGB1. Immunohistochemical staining and immunofluorescence assays showed that conjunctival cells from pterygia had an increased amount of cytoplasmic HMGB1 compared to the control (Figure 5b). Notably, the western blot analysis showed that the mean HMGB1 level in cytoplasmic fractions of conjunctival tissues from the five patients was significantly increased compared to that of control conjunctival tissues (Figure 5c). To measure the extracellular secretion of HMGB1, we collected the media from cultured pterygium and control tissues after 8 h of ELISA. HMGB1 levels in the culture supernatant were increased in pterygium tissues (Figure 5d). In addition, immunohistochemistry also showed increased expression of CXCL12, one of the strong chemotactic factors induced by HMGB1^14^ in the pterygia compared to normal conjunctiva (Supplementary Figure S2).

Taken together, these data present that ROS production caused by UV induces chronic inflammation through HMGB1 translocation in the pterygial tissue and UV-induced ROS is involved in the HMGB1-mediated pterygium formation.^33, 34^

## Discussion

In our study, we confirmed that HMGB1 was translocated in the cytoplasm after UV treatment on ocular surface tissue. Antioxidant treatment was found to reduce HMGB1 translocation while attenuating conjunctival inflammation by UV-induced HMGB1 and decrease the infiltration of leukocytes to the inflammatory site. Pretreatment with the HMGB1 inhibitors sRAGE and Box A of HMGB1 decreased UV-induced leukocyte migration. There have been several studies regarding HMGB1 stimulating the motility of various cell types such as fibroblasts, dendritic cells, macrophages, smooth muscle cells, and, most recently, neutrophils.^35, 36^ As an alarmin, treatment with a high concentration of recombinant HMGB1 protein (5 *μ*g/ml or 300 nM) was confirmed to attract inflammatory cells through interactions with RAGE^37^ and CXCR4.^14^ In chronic inflammatory disorders, such as rheumatoid arthritis, a high amount of HMGB1 was reported in the target organ where it induced leukocyte recruitment, resulting tissue damage.^38^

We also demonstrated that UV-induced ROS generation and oxidative stress caused HMGB1 extracellular secretion. It was confirmed by pretreatment with an ROS quencher that decreased UV-induced HMGB1 secretion. We hypothesize that UV exposure induces the translocation of HMGB1 protein to the extracellular environment where it recruits leukocytes to the UV-exposed site causing ocular surface inflammation. We could assume that the concentration of locally secreted HMGB1 might be relatively high because small amounts of HMGB1 protein did not attract neutrophils.^37^ Normal human corneal cells express TLRs 1–10 while normal human uvea, sclera, retina, and conjunctiva express mRNA associated with TLR4 and its lipopolysaccharide receptor complex.^39, 40, 41^ In this study, we could not specifically identify the receptors related to the chemotactic effect of HMGB1. The exact receptors that HMGB1 uses to induce its inflammatory effects therefore require further evaluation. In addition, it would be worthwhile to study the oxidative conditions of secreted HMGB1 SH residues in pterygium samples because only the fully reduced form of HMGB1, in which all three cysteines are in a thiol state, can function as a chemoattractant.^42^

As a model disease, pterygium, the UV-induced chronic ocular surface inflammation, we found increased HMGB1 and ROS compared to the control samples. The oxidative condition is one of the major factors affecting the localization and function of HMGB1.^5^ The study demonstrated reduction in the movement of HMGB1 caused by mitochondrial ROS inhibitor, and it suggests possible interaction between HMGB1 and mitochondrial system. HMGB1 is known to be located in the mitochondria in HUVEC cells, and high-mobility group A1 (HMGA1) protein, a nuclear transcription factor belonged to the HMG family, was also found to be in the mitochondria in several cell lines.^43, 44^ Recently, there was a study confirming that extracellular HMGB1 protein induces the phosphorylation of RAGE, which then accumulates in mitochondria.^45^ We hypothesize that cytosolic HMGB1 might also be located in mitochondria, and that mitochondrial electron transport systems might influence the oxidative potential and location of HMGB1. In addition, it is possible that HMGB1 secreted to the extracellular environment might interact with various receptors, promoting mitochondrial synthesis or mitochondrial biogenetics.

UV irradiation can upregulate the expression of other damage-associated molecular pattern (DAMP) molecules such as heat shock proteins, and these molecules also depict chemotactic effects.^46, 47, 48^ Moreover, other DAMP molecules could be target molecules in UV-induced ocular surface changes, including pterygium formation. We could confirm the increase in IL-8 in our Chang cell study. It is already proven that IL-8 is an UV-mediated cytokine in the pterygium and human pterygium epithelial cell through the previous study.^23^ It is possible that HMGB1 might induce the secretion of these cytokines through interactions with other membranous receptors. This study proved a profound role of HMGB1 for UV-induced inflammation in conjunctive. However, lack of detailed experiments regarding mechanisms or pathways of HMGB1 release still remains as a limitation of the study.

In conclusion, we report that ocular surface inflammation caused by UV exposure results HMGB1 translocation, and it is originated from ROS generation. Our findings were identical to the previously unknown mechanism of ocular surface inflammation, suggesting a new medical therapeutic target for UV-induced changes regulating its development.

## Materials and Methods

### Mice conjunctival UV exposure model

Six- to eight-week-old female BALB/c mice were purchased from Charles River Laboratories (Orient Bio Inc., Sungnam, Korea). The conjunctivas of BALB/c mice were exposed to UV radiation (311 nm wavelength) at a single dose of 100 mJ/cm^2^ using a UV lamp (Philips, Eindhoven, Holland). To block the effect of HMGB1, mice were injected intraperitoneally with 100 *μ*g of sRAGE 15 min before UV radiation or with 150 *μ*g of HMGB1 A box 1 h before and after UV radiation. For inhibition of ROS production, mice were intraperitoneally injected with an ROS scavenger consisting of 125 mM NAC (Sigma-Aldrich, St. Louis, MO, USA) 2 h before UV radiation or with 135 *μ*M Mito-Tempo (Enzo Life Sciences) 24 h and 1 h before UV radiation, respectively, and were then killed 24 h after UV radiation.

### Cell culture and DNA transfection

Human conjunctival epithelial Chang cells were purchased from the Korean Cell Line Bank (KCLB, Seoul, South Korea) and cultured at 37 °C under 5% CO_2_ in RPMI 1640 medium containing 2 mM l-glutamine, 25 mM HEPES, 25 mM NaHCO_3_, 10% FBS, 100 U/ml penicillin, and 100 *μ*g/ml streptomycin (Gibco, Invitrogen, Carlsbad, CA, USA). Transient transfection of the HMGB1-GFP plasmid was performed using FuGENE HD (Promega, Mannheim, Germany).^49^

### Protein preparation

Recombinant wild-type HMGB1 and mouse sRAGE-Fc protein were produced in CHO cells (A&R Therapeutics, Daejeon, Republic of Korea) and HMGB1 box A (aa 1–79) was produced in *E. coli* BL21 (DE3) pLysE followed by purification using the Ni^2+^-NTA system, Sephadex G75 medium, and ion-exchange column.^50^ Endotoxin was removed using Triton X-114 (Calbiochem, La Jolla, CA, USA).^51^

### UV radiation

Chang cells were cultured in a 100-mm culture dish and grown until they reached semi-confluence. The medium was aspirated and the cells were washed three times with cold PBS prior to UV irradiation. UV intensity was monitored and calibrated before each experiment with the aid of a model-500-C radiometer (National Biological Corporation, Twinsborg, OH, USA). After UV exposure, cells were re-washed three times with cold PBS and incubated for further 8 h in culture medium.

### Treatment of ROS scavengers and detection of ROS

To detect the ROS generated by UV radiation, Chang cells were irradiated with UV, and 5 *μ*M of dichlorodihydrofluorescein diacetate (DC-FDA; Molecular Probes Inc., Eugene, OR, USA) was added to the culture medium and incubated for 30 min. The UV-generated ROS level was observed by FV1000 confocal microscopy (Olympus, Tokyo, Japan). To observe UV-induced cytoplasmic HMGB1 translocation, Chang cells were transiently transfected with an HMGB1-GFP plasmid and treated with 10 mM NAC or 50 nM Mito-Tempo (Enzo Life Sciences) for 1 h followed by UV radiation. Cells were cultured for further 8 h after UV radiation after which GFP-tagged HMGB1 protein or endogenous HMGB1 were immunostained using an anti-HMGB1 antibody (Ab) and observed by confocal microscopy. We counted 100 GFP-positive cells in multiple random visual fields in each condition and compared the results to the number of cytoplasmic HMGB1-containing cells for nucleo-cytoplasmic translocation.

### Nuclear and cytoplasmic extraction

The extraction of nuclear and cytoplasmic fractions was performed using a nuclear/cytosol fractionation kit (BioVision, Mountain View, CA, USA) following the manufacturer's protocol. Briefly, cells were harvested and cytoplasmic extraction buffer A mix (containing DTT and protease inhibitors) and CEB-B were added consecutively to extract the cytoplasmic proteins. After separating the cytoplasmic extract, a nuclear extraction buffer (NEB) mix (containing DTT and protease inhibitors) was added and the nuclear extract was separated by centrifugation.

### Western blot analysis

Chang cells were lysed using Pro-Prep protein extraction solution (Intron, Seongnam, Korea) that included a mixture of protease inhibitors (Sigma-Aldrich). The protein concentration was determined by using the bicinchoninic acid (BCA) assay (Pierce Thermo Scientific, Rockford, IL, USA). Protein samples were loaded on a 12% SDS-PAGE gel and transferred to nitrocellulose membranes. Western blot analysis was performed using primary Abs against HMGB1 (Abcam, Cambridge, UK), *β*-actin (Santa Cruz Biotechnology Inc, Santa Cruz, CA, USA), LDH (Santa Cruz Biotechnology Inc), and *α*-tubulin (Santa Cruz Biotechnology Inc), and HRP-labeled goat anti-rabbit or goat anti-mouse secondary Abs (Jackson Immuno Research Labs Inc, West Grove, PA, USA). ECL was used to reveal the signals (Thermo Scientific, Rockford, IL, USA), and band intensities were measured using the ImageJ program (NIH, Bethesda, MD, USA). To compare the HMGB1 secretion levels, cells were seeded into 60-mm plates and maintained in OPTI-MEM (Gibco, Invitrogen). UV was irradiated when the cells were semi-confluent, after which they were incubated for further 8 h. Cell culture supernatants were then collected and concentrated using a centrifugal filter unit (Millipore, Billerica, MA, USA) followed by western blot analysis using an anti-HMGB1 Ab.

### ELISA to detect secretory HMGB1 levels

Secretory HMGB1 levels in the culture supernatant of pterygium and control tissues were determined by ELISA with reference to the standard curve for purified HMGB1 (Shino-Test, Tokyo, Japan), obtained with serial dilutions. The concentrations of the purified HMGB1 standard ranged from 0 to 160 ng/ml.

### cDNA synthesis and real-time quantitative PCR

The human conjunctiva epithelial Chang cells at different time points following UVB irradiation were collected. Total RNA was extracted with TRIzol reagent (Invitrogen, Carlsbad, CA, USA), and 5 *μ*g of RNA was converted into cDNA using Superscript II reverse transcriptase with oligo (dT) primer (Thermo Scientific). The products were used as a template for each chemokine-specific real-time PCR set for the amplification of MIP-1, MCP-1, IL-17, IL-8, and HMGB1. Quantitative PCR amplifications were performed using SYBR Green (Thermo Scientific) in a Chromo4 real-time thermocycler (Bio-Rad, Hercules, CA, USA).

### Detection of 8-hydroxy-2-deoxy guanosine concentration

Chang cells were harvested and DNA was isolated using a genomic DNA extraction kit (Qiagen, Valencia, CA, USA). The amount of 8-hydroxy-2-deoxy guanosine was determined using an OxiSelect oxidative DNA damage ELISA kit (Cell Biolabs, San Diego, CA, USA) according to the manufacturer's instructions.

### Histological analysis

The conjunctivas of BALB/c mice were exposed to UV (311 nm wavelength) at a single dose of 100 mJ/cm^2^ using a UV lamp (Philips). For histological analysis, 24 h after UV treatment, whole eyes were enucleated and were fixed in 1% paraformaldehyde followed by routine paraffin embedding. Sections were stained with H&E, and the number of infiltrated leukocytes was counted by light microscopy at the bulbar and fornical regions of the conjunctiva field using a magnification of × 200 for each H&E-stained slide.

Frozen sections were also prepared for additional mouse experiments as well as with human pterygium tissues. Whole eyes were enucleated from mice 24 h after the conjunctiva were exposed to UV (single dose of 100 mJ/cm^2^) and were frozen after embedding in OCT solution. Frozen 6-*μ*m-thick sections were blocked with 2% BSA in PBS for 45 min and incubated overnight at 4 °C with the following primary Abs: rabbit anti-HMGB1 (Abcam), rabbit anti-mouse F4/80 (eBioscience, San Diego, CA,USA), and rat anti-mouse Gr-1 (BD Pharmingen, eBioscience); an isotype-matched Ab was used as a control. The following day, the sections were incubated with Alexa Fluor 488- or Alexa Fluor 594-conjugated secondary Ab at room temperature for 1 h. Sections were mounted with DAPI-containing mounting medium, and were examined under a confocal microscope.

For immunohistochemical staining, the 4-*μ*m paraffin tissue sections were de-paraffinized, rehydrated, and treated with an antigen retrieval solution (10 mM sodium citrate buffer, pH 6.0). The sections were incubated with a dilution of 1 : 1000 rabbit anti-human HMGB1 Ab (Abcam) and mouse monoclonal anti-human CXCL12 (R&D Systems, Minneapolis, MN, USA) overnight at 4 °C, and then incubated with polymer-HRP Ab (DAKO, Glostrup, Denmark) for 20 min at room temperature according to manufacturer's instructions. Finally, tissue sections were incubated with DAB (Sigma-Aldrich) until a brown color developed, before the sections were counterstained with hematoxylin.

### Immunofluorescence and confocal imaging

Cells were cultured in a four-chamber slide (Nunc, Naperville, IL, USA) and fixed with 4% paraformaldehyde for 20 min at RT. After fixation, cells were washed three times with cold PBS and permeabilized with 0.2% Triton X-100 for 10 min, and then blocked with 1% BSA-PBS for 1 h at RT. Cells were then incubated overnight at 4 °C with primary Abs that had been diluted in 1% BSA-PBS. The cells were washed three times with cold PBS, and Alexa Fluor 488- or Alexa Fluor 594-conjugated secondary Abs (Invitrogen) were added for 1 h. The cells were washed three times with cold PBS and mounted with Vectashield mounting solution (Vector Laboratories, Burlingame, CA, USA) and observed under a confocal microscope.

### Statistical analysis

The statistical analysis was performed by using the Student's *t*-test. All data are presented as mean±S.E.

### Ethical declaration

The Institutional Review Board of Yonsei University College of Medicine approved this study, and the study adhered to the tenets of the Declaration of Helsinki (Yonsei University Health System, No. 4-2013-0656).

## Abbreviations

DAMP: damage-associated molecular pattern
HMGB1: high-mobility group box 1 protein
ROS: reactive oxygen species
