# Supplementary figures and images for: HMGB1 in the pathogenesis of ultraviolet-induced ocular surface inflammation
Source: Cell Death Dis. 2015 Aug 27;6(8):e1863–. doi: 10.1038/cddis.2015.199 (PMC4558494; doi:10.1038/cddis.2015.199)

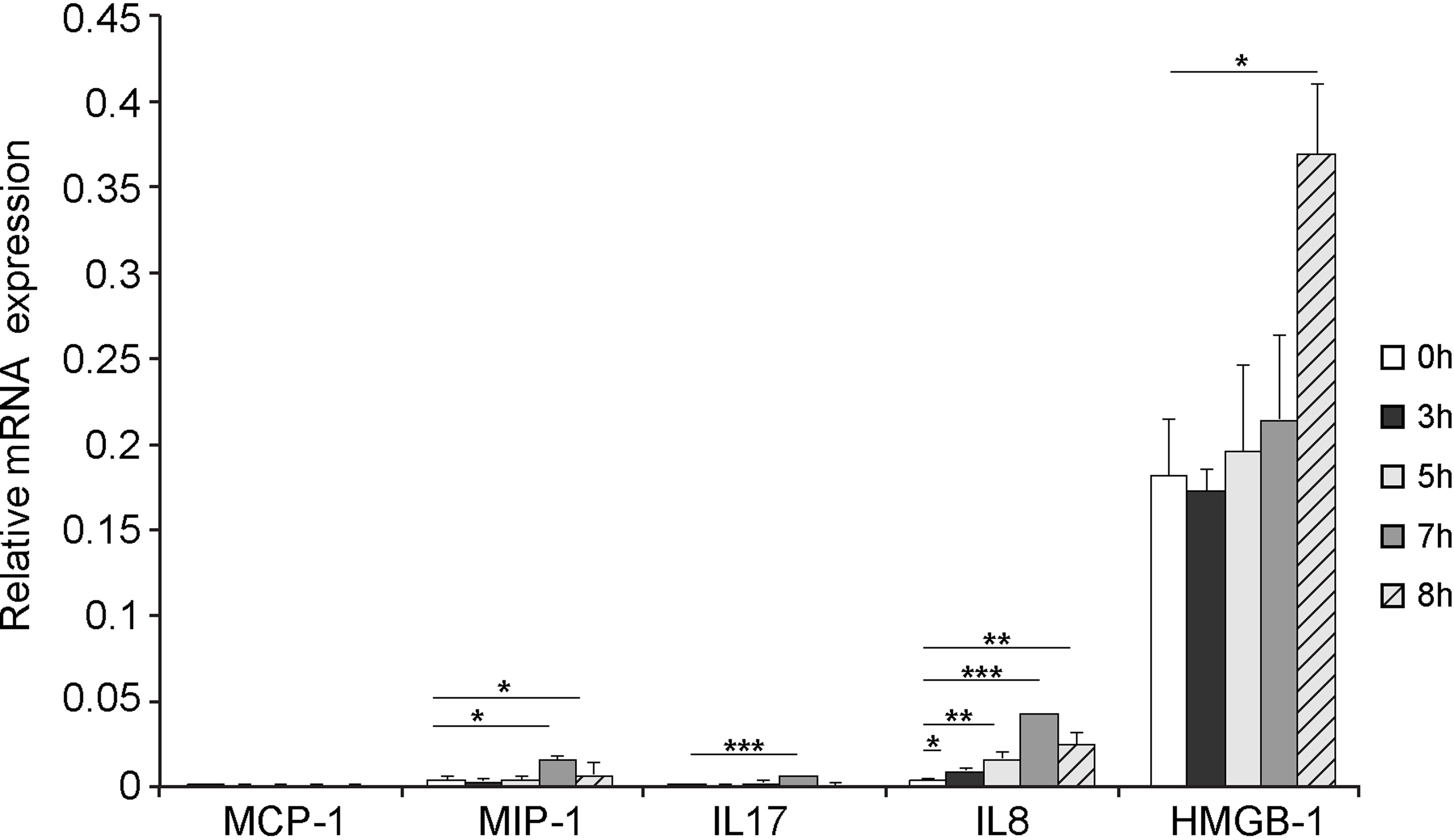

Supplement: Supplementary Figure 1 [file cddis2015199x1.tif]

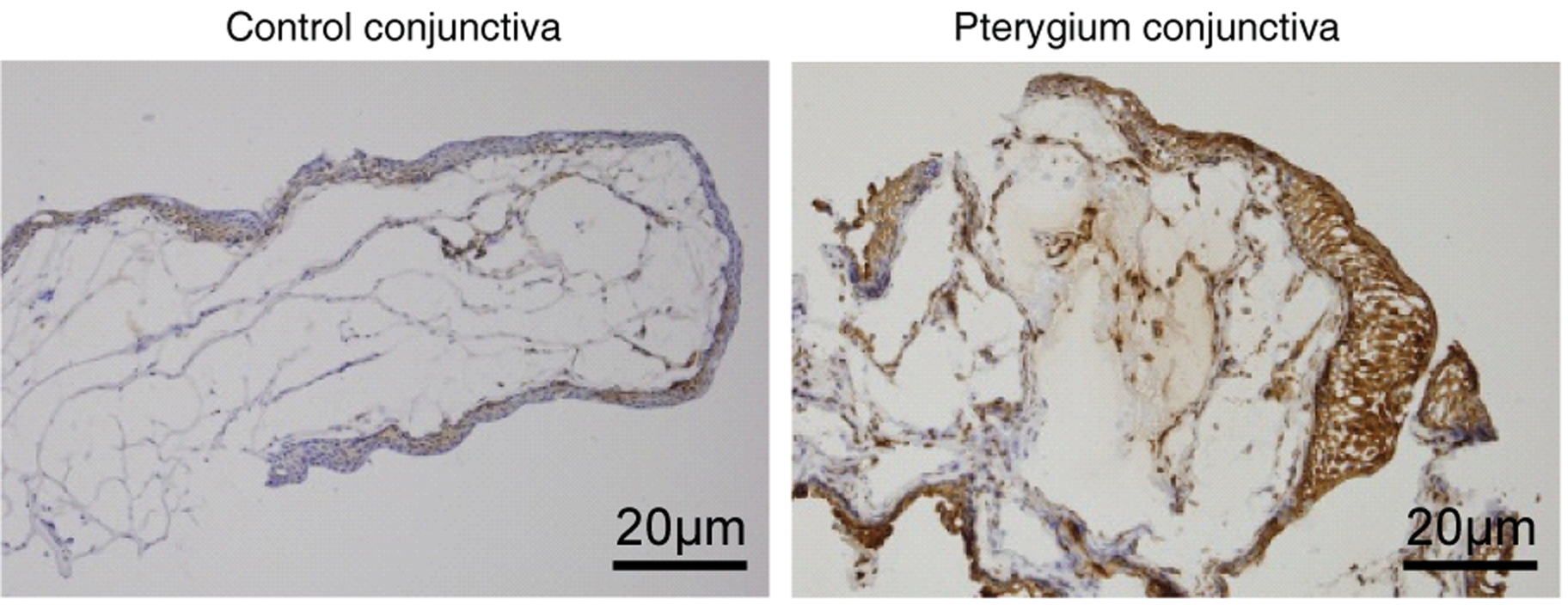

Supplement: Supplementary Figure 2 [file cddis2015199x2.tif]
