# Supplementary material for: HMGB1 in the pathogenesis of ultraviolet-induced ocular surface inflammation
Source: Cell Death Dis. 2015 Aug 27;6(8):e1863–. doi: 10.1038/cddis.2015.199 (PMC4558494; doi:10.1038/cddis.2015.199)
Supplement: Supplementary Figure Legends [file cddis2015199x3.doc]

**Supplementary Figure legends**

**Figure S1.** **Cytokine expressions in human conjunctiva epithelial Chang cell after UVB-irradiation.** Human conjunctiva epithelial chang cell were collected at 0, 3, 5, 7, and 8 h after UVB irradiation. mRNA expression levels of MCP-1, MIP-1, IL-17, IL-8, and HMGB1 were determined by real-time PCR. **P* < 0.05, ** *P* < 0.01, *** *P* < 0.001.

**Figure S2. CXCL12 expression in human pterygium tissue.**

Human pterygial tissues were surgically resected from five patients and immunohistochemical assays were performed for CXCL12. Control conjunctival tissues from the superior bulbar conjunctiva of the same patients were used for comparison.
